# Supplementary material for: Residue proximity information and protein model discrimination using saturation-suppressor mutagenesis
Source: eLife. 2015 Dec 30;4:e09532. doi: 10.7554/eLife.09532 (PMC4758949; doi:10.7554/eLife.09532)
Supplement: Supplementary File 1. — DOI: http://dx.doi.org/10.7554/eLife.09532.026 [file elife-09532-supp1.docx]

**Supplementary File 1**. Summary of sort details for different libraries of CcdB. PIM refers to the parent inactive mutant.

| Sr. no. | PIM | Sort Round # | Input | Output | [Gyrase] (nM) | Relative population of PIM in sorted gate (%) | Relative library population in sorted gate (%) | Growth temperature (^o^C) |
| --- | --- | --- | --- | --- | --- | --- | --- | --- |
| 1 | V5F | 1  2  3 | V5F_Lib  V5F_Lib1  V5F_Lib2 | V5F_Lib1  V5F_Lib2  V5F_Lib3 | 16,000  4,000  64 | 0.3  0  0 | 0.2  0.3  0.7 | 20 |
| 2 | V18W | 1  2  3  4  5  6 | V18W_Lib  V18W_Lib1  V18W_Lib2  V18W_Lib3  V18W_Lib4  V18W_Lib5 | V18W_Lib1  V18W_Lib2  V18W_Lib3  V18W_Lib4  V18W_Lib5  V18W_Lib6 | 16,000  8,000  4,000  1,000  16  4 | 0.7  0.9  0.1  0.6  0.1  0 | 0.6  0.8  1.5  0.5  0.9  0.8 | 20 |
| 3 | V20F | 1 | V20F_Lib | V20F_Lib1 | 8,000 | 2.2 | 0.3 | 20 |
|  |  | 2 | V20F_Lib1 | V20F_Lib2 | 2,000 | 1.6 | 0.8 |  |
|  |  | 3 | V20F_Lib2 | V20F_Lib3 | 256 | 0.7 | 0.4 |  |
|  |  | 4 | V20F_Lib3 | V20F_Lib4 | 16 | 0 | 0.6 |  |
|  |  | 5 | V20F_Lib4 | V20F_Lib5 | 4 | 0 | 0.5 |  |
| 4 | L36A | 1 | L36A_Lib | L36A_Lib1 | 4 | 0 | 0.6 | 30 |
|  |  | 2 | L36A_Lib1 | L36A_Lib2 | 2 | 0.1 | 0.8 |  |
|  |  | 3 | L36A_Lib2 | L36A_Lib3 | 1 | 0.3 | 0.8 |  |
|  |  | 4 | L36A_Lib3 | L36A_Lib4 | 0.25 | 0 | 1.9 |  |
| 5 | L83S | 1 | L83S_Lib | L83S_Lib1 | 4 | 6.8 | 0.4 | 30 |
|  |  | 2 | L83S_Lib1 | L83S_Lib2 | 1 | 0.1 | 0.3 |  |
|  |  | 3 | L83S_Lib2 | L83S_Lib3 | 0.25 | 0 | 0.7 |  |
