# Supplementary material for: Residue proximity information and protein model discrimination using saturation-suppressor mutagenesis
Source: eLife. 2015 Dec 30;4:e09532. doi: 10.7554/eLife.09532 (PMC4758949; doi:10.7554/eLife.09532)
Supplement: Supplementary File 2. — DOI: http://dx.doi.org/10.7554/eLife.09532.027 [file elife-09532-supp2.docx]

**Supplementary File 2**. Relative expression, binding and stabilities of parent inactive mutants and their suppressors in the case of CcdB

| Protein | K_d_ (nM) determined by FACS | Saturation MFI^a^ (surface expression) | Saturation MFI^a^ (binding) | T_m_^b^ (^o^C) in absence of CcdA | T_m_^b^ (^o^C) in presence of CcdA |
| --- | --- | --- | --- | --- | --- |
| WT | 0.3 ±0.1 | 1280 ±181 | 14225 ±1883 | 66.8 ± 1.0 | 78.0 ± 0.1 |
| R10G | 5.3±0.7 | 1676 ±56 | 13497 ±90 | 74.8 ± 0.2 | 76.1 ± 0.1 |
|  |  |  |  |  |  |
| V5F^c^ | >120 | 363 ±7 | 153 ±7 | ^d^ | ^d^ |
| V5F/A81G/M63T^c^ | 5.6 ± 0.1 | 436 ±42 | 4572 ±806 | ^e^ | ^e^ |
|  |  |  |  |  |  |
| V18W^c^ | >258 | 291±13 | 153 ±26 | 41.9 ±0.5 | 64.4 ± 0.5 |
| V18W/M63T^c^ | 2.3 ± 0.5 | 407 ±1 | 1089 ±112 | 41.8 ± 0.4 | 72.4 |
| V18W/I90V^c^ | 67.9 ± 8.3 | 454 ±37 | 920 ±202 | ^d^ | ^d^ |
|  |  |  |  |  |  |
| V20F^c^ | >988 | 667 ±18 | 264 ±21 | ^d^ | ^d^ |
| V20F/E11R^c^ | 16.7 ± 0.9 | 577 ±41 | 1011 ±513 | ^d^ | ^d^ |
|  |  |  |  |  |  |
| L36A | 1.9 ± 1.2 | 316 ±7 | 2682 | 47.1±0.3 | 70.3 ± 0.7 |
| L36A/M63L | 0.6 | 1106 ± 67 | 10642 ±23 | 55 ± 0.8 | 75.9 ± 0.1 |
| L36A/R10G | 1.2 ± 0.4 | 867 ±67 | 12040 ±1088 | 55.1 ± 0.4 | 64.0 ± 0.4 |
|  |  |  |  |  |  |
| L83S | 2.1 ± 0.9 | 253 ±5 | 2175 ±130 | 46.0 ± 0.6 | 70.3 ± 0.1 |
| L83S/V54L | 0.4 ± 0.3 | 843 ±21 | 13615 ±151 | 52.5 ± 0.6 | 74.2 ± 0.3 |

^a^MFI corresponds to Mean Fluorescence Intensity observed from FACS analysis.

^b^T_m_ corresponds to melting temperature of purified proteins monitored by thermal denaturation (see Materials and Methods) of 4µM of CcdB (toxin) protein in presence/absence of 20µM CcdA (antitoxin) peptide (residues 46-72). The presence of ligand, CcdA shifts the unfolding equilibrium towards the folded fraction of CcdB, resulting in an increased T_m_ than when monitored in its absence.

^c^The proteins have been induced at 20^o^C prior to sort and analysis by FACS. The remaining proteins have been induced at 30^o^C.

^d^Protein could not be purified, and hence T_m_ was not measured. V5F, V20F, V20F/E11R, V18W/I90V CcdB mutants were unstable and could not be purified due to either low expression and/or inability to bind to CcdA peptide (immobilized to the column for purification) (see Materials and Methods).

^e^The protein was purified but not subjected to thermal denaturation due to difficulty in purifying its parent inactive mutant. Thus, comparative stability analysis of the pair could not be carried out.

± indicates standard deviation from two independent experiments.
