# Supplementary material for: Residue proximity information and protein model discrimination using saturation-suppressor mutagenesis
Source: eLife. 2015 Dec 30;4:e09532. doi: 10.7554/eLife.09532 (PMC4758949; doi:10.7554/eLife.09532)
Supplement: Supplementary File 3. — Experimentally observed (Parent inactive mutant, suppressor) pairs are indicated in bold. Main chain atoms and hydrogen atoms are not considered in the calculations. DOI: http://dx.doi.org/10.7554/eLife.09532.028 [file elife-09532-supp3.docx]

**Supplementary File 3.** Sidechain-sidechain centroid distances and shortest distances between the listed residue pairs for putative differential contacts between DgkA X-ray and NMR structures. Experimentally observed (Parent inactive mutant, suppressor) pairs are indicated in bold.

| **Parent inactive mutant (X)** | **Differential** | **Centroid-centroid Distance (Å)^b^** | | **Shortest Distance (Å)^c^** | |
| --- | --- | --- | --- | --- | --- |
|  | **Contact Residues^a^** |  |  |  | |
|  | **(Y)** | **X-ray** | **NMR^d^** | **X-ray** | **NMR^d^** |
|  |  |  |  |  |  |
| **62** | **41** | **3.8** | **8.4** | **3.6** | **8** |
| 62 | 108 | 4.9 | 11.4 | 4.4 | 10.3 |
| 62 | 112 | 5.8 | 15.8 | 3.1 | 13 |
| 62 | 102 | 14.9 | 6.5 | 14.1 | 5.1 |
| 62 | 103 | 10.9 | 3.9 | 9.2 | 3.5 |
| 66 | 31 | 5.2 | 10.8 | 3.7 | 9.2 |
| **66** | **35** | **4.8** | **10.4** | **3.6** | **8.4** |
| **66** | **38** | **3.8** | **9.5** | **3.5** | **7.8** |
| 66 | 99 | 8.5 | 3.7 | 7.5 | 3.1 |
| 67 | 100 | 5.1 | 10.8 | 4.2 | 10.1 |
| **67** | **103** | **5.4** | **12.4** | **3.6** | **9.8** |
| **67** | **104** | **4.7** | **16.1** | **3.8** | **15** |
| 67^e^ | 31^e^ | 10.5 | 8 | 8.3 | 5.7 |
| 67 | 34 | 9.9 | 5 | 7.8 | 3 |
| **68** | **100** | **5** | **12.9** | **3.9** | **11.7** |
| 68 | 101 | 5.1 | 13.8 | 3.6 | 12.2 |
| 68 | 104 | 4.1 | 16.6 | 4 | 16.3 |
| 68 | 31 | 11.2 | 6.1 | 9.5 | 4.6 |
| 68 | 35 | 13 | 5.3 | 12.5 | 4.5 |
| 112 | 41 | 5.2 | 10.2 | 3.5 | 8.8 |
| 112 | 44 | 6.9 | 8.4 | 4.1 | 5.7 |
| 112 | 58 | 4.2 | 8.4 | 3.5 | 8.3 |
| 112 | 61 | 5.5 | 15.2 | 3.3 | 13.7 |

^a^Contact present in either X-ray or NMR structure. Contact defined by sidechain-sidechain centroid distance≤7Å.

^b^Distance between side chain centroids of residues X and Y

^c^Shortest distance between residues X and Y
^d^Closest distance amongst the distances calculated for all 16 poses of the NMR structure

^e^For the residue pair 67 – 31, the distances in both the X-ray and NMR structures is >7Å, in the case of the NMR structure, the side chains point towards each other and hence it was shortlisted as an NMR contact.
